# *In vivo* assessment of broccoli extract based hydrogel in mitigating DMBA induced breast cancer in female mice

**DOI:** 10.3389/fonc.2026.1947816

**Published:** 2026-09-11

**Authors:** Kajal Parashar, Minhaj Ahmad Khan, Meenakshi Verma, Pratibha Pandey, Shailendra Thapliyal, Fahad Khan

**Affiliations:** 1 Department of Zoology, School of Bioengineering & Biosciences, Lovely Professional University, Phagwara, Punjab, India; 2 School of Allied Health Sciences (AHS), CGC University, Mohali, Punjab, India; 3 University Centre for Research and Development, Chandigarh University, Mohali, Punjab, India; 4 Uttaranchal Institute of Technology, Uttaranchal University, Dehradun, Uttarakhand, India; 5 Department of Community Medicine, Saveetha Medical College and Hospitals, Saveetha Institute of Medical and Technical Sciences, Chennai, Tamil Nadu, India

**Keywords:** breast cancer, DMBA, drug delivery, hydrogel, mice

## Abstract

**Introduction:**

Breast cancer remains a major global health burden, underscoring the need for safer and more effective therapeutic strategies. Broccoli-derived bioactive compounds, particularly sulforaphane, exhibit antioxidant, anti-inflammatory, pro-apoptotic, and antiproliferative activities. However, their therapeutic translation may be limited by inadequate localization and systemic delivery. This study aimed to develop a broccoli extract-loaded hydrogel as a novel localized drug delivery platform for breast cancer therapy.

**Methods:**

The therapeutic efficacy of the broccoli extract-loaded hydrogel was evaluated in a DMBA-induced mammary tumor model using female BALB/c mice. Treatment-induced alterations in oxidative stress and antioxidant defense were assessed by quantifying malondialdehyde (MDA), nitrite, superoxide dismutase (SOD), and glutathione (GSH). Histopathological analyses of mammary gland and liver tissues were performed to further evaluate tumor-associated tissue alterations and treatment-mediated protection.

**Results and Discussion:**

Administration of the broccoli extract-loaded hydrogel markedly attenuated tumor-associated oxidative stress, as demonstrated by reduced MDA and nitrite levels, while restoring antioxidant defense through improved SOD and GSH status compared with untreated tumor-bearing controls. Histopathological findings further supported the protective effects of treatment, with improved tissue architecture in mammary gland and liver. The enhanced therapeutic response may be attributed to localized and sustained delivery of broccoli-derived phytochemicals from the hydrogel matrix, facilitating prolonged exposure at the target site while potentially limiting systemic toxicity. Collectively, these findings establish broccoli extract-loaded hydrogel as a promising natural, localized, and potentially safer therapeutic platform for breast cancer management and provide a foundation for further translational investigation.

## 1 Introduction

Breast cancer (BC) is the 2nd most commonly diagnosed cancer worldwide and is responsible for a large portion of cancer-related deaths. The most prevalent malignancy among women globally is breast cancer (1). For many years, the leading cause of cancer-related deaths and morbidities among women worldwide has been female BC. In 2020, there were an anticipated 2.3 million new cases & 685,000 deaths from female breast cancer globally, accounting for one in four cancer cases along with one in six cancer-related deaths among women (2). It has been shown that genetic mutations & family history account for 5% to 10% of breast cancers, whereas potentially modifiable variables account for 20% to 30% of cases (3).

In clinical practice, human epidermal growth factor receptor 2 (HER2), progesterone receptor (PR), and estrogen receptor (ER) are important indicators used for clinical trial inclusion, therapy selection, and prognostic prediction. Luminal A, Luminal B, HER2-enriched, and triple-negative breast cancer (TNBC) are the four subtypes of breast cancer that are identified by immunohistochemistry staining of these markers (4). The high incidence suggested that female-specific factors associated with reproductive characteristics, such as early age at menarche, later age at menopause, advanced age at first birth, fewer children, less breastfeeding, hormonal menopausal therapy, oral contraceptive, along with breast density, also collectively contributed to a significant number of BC cases. Approximately 30% of BC cases worldwide are attributable to modifiable risk factors, like as excess body weight, physical inactivity, and consumption of alcohol, and may therefore be preventable (5).

The high number of BC cases is mostly due to the rapid ageing and population growth, the significant changes in female reproductive traits brought about by socioeconomic development, and the fact that most transitioning nations have not yet implemented efficient BC screening programs. Numerous studies have already examined the worldwide burden of breast cancer using GLOBOCAN 2020 or older data. But as of right now, there aren’t many assessments that use the most recent GLOBOCAN 2022 data to evaluate the illness burden (6). The GLOBOCAN 2022 database (https://gco.iarc.fr/) included data on new cases and deaths, age-standardized incidence and mortality rates, predicted yearly percentage changes, and demographic predictions up to 2050. The GLOBOCAN database is created by the IARC under the World Health Organisation (WHO) by aggregating and combining country-specific cancer burden data supplied by nations worldwide, following consistent standards (7), (8).

Breast cancer patients can receive a variety of traditional treatment options, including hormonal therapies, monoclonal antibody therapies, immunotherapy, small molecular inhibitors, radiotherapy, and chemo-radiotherapies (such as adjuvant chemotherapies as well as neoadjuvant therapy) (9). These therapeutic approaches do, however, come with risks and adverse effects. Therefore, to minimize the drawbacks associated with breast cancer patients, including growing resistance to traditional therapies, side effects, and the toxicities of current treatment modalities, new techniques and methods are required (10). Traditional therapies for breast cancer, such as radiation therapy and chemotherapy, frequently have poor selectivity and can damage healthy organs and tissues in addition to the cancer cells they target. The effectiveness of therapy may be limited, patients’ quality of life may be negatively impacted, and serious adverse effects may result from this systemic toxicity. The capacity of cancer cells to adapt and become resistant to immunotherapy, targeted treatments, and chemotherapy is remarkable. This acquired resistance increases the risk of illness progression and recurrence and compromises the efficacy of treatment plans (11). Furthermore, many people throughout the world lack easy access to and financial means for synthetic medications (12).

The well-known green vegetable “broccoli” (*Brassica oleracea* var. *italica*) is a staple of the Mediterranean diet because of its many health advantages. Broccoli’s possible anticancer effect is one of the main bioactive effects that have been identified. Broccoli is one of the vegetables in the Brassicaceae family that is becoming more and more popular since numerous epidemiological studies have shown that vegetables high in certain phytochemicals are linked to a lower incidence of breast cancer and other cancers (13). Because of their potential to prevent cancer, glucosinolates are one of the most significant phytochemicals among all the bioactive components of broccoli. It is noteworthy to add that over 200 glucosinolates are currently identified thus far. Sulforaphane, an organosulfur molecule that is abundant in broccoli and a member of the isothiocyanate category of phytoconstituents (14).

There have been few studies on broccoli’s ability to prevent cancer in people to date. Most studies on broccoli extract’s anti-cancer properties concentrated on the properties of sulforaphane, a compound found in cruciferous plants (15). Chemopreventive agents have been included in combination treatment strategies to achieve a therapeutic harmony between individual drugs while limiting systemic toxicity induced by these therapies. This avoids the complications related to standard cancer treatments, such as chemotherapy. Bioactive phytochemicals produced from broccoli seem to be particularly useful in the treatment of breast cancer. Because of their ability to prevent breast cancer, glucosinolates are one of the most significant phytochemicals among all the bioactive components of broccoli. Organosulfur compound sulforaphane (SFN) *(1-isothiocyanato-4-methylsulfinylbutane*), which is abundant in broccoli and is the major bioactive anticancerous compound in it (16). It is further anticipated that herbal and traditional medicine is safer and less expensive, and that it has fewer unintended side effects than most modern medications. Herbal ingredients have few negative effects on humans, but they also provide the body with beneficial minerals and nutrients (17). SFN is a sulfur-containing glucosinolate found particularly in cruciferous vegetables such as broccoli. Sulforaphane is a key anti-cancer phytochemical due to its ability to activate cellular detoxification and antioxidant pathways, notably NRF2 (18).

Therefore, to deliver these phytotherapeutics, a sustained and controlled delivery of drugs is needed for the breast cancer treatment. By regulating the pace, time frame, and distribution of drug release, drug delivery systems are intended to enhance the pharmacokinetics (drug movement) and pharmacodynamics (drug biological reaction and associated mechanism) of medications. A range of materials, including as metals, lipids, and polymers, can be used to create drug delivery systems. Depending on the medicine being given, the intended location of action, and the preferred mechanism of action, a substance like hydrogel carrier must be chosen and its synthesized (19).

Hydrogels are three-dimensional networks of highly hydrated polymers that may encapsulate and release medications over an extended period of time, making them appropriate for therapies requiring targeted or prolonged release (20). A hydrogel is a three-dimensional network substance made up of crosslinked side chains with hydrophilic or hydrophobic groups and a polymeric backbone. Hydrogels, with their high-water content, variable physical characteristics, elastic modulus, flexibility, reversible swelling, & multifunctionality, are promising biomaterials for use in biomedicine and bioengineering (21). Hydrogels’ special porosity structure facilitates the free diffusion of ions and water molecules and increases their reactivity to outside stimuli. This aids in the creation of intelligent hydrogels that can lessen harm to healthy tissues and release medications in response to internal or external stimuli (22).

Hydrogels have minimal toxicity, swelling behavior, and biocompatibility, which makes them good options for biological uses, including medication administration. Drug development faces significant challenges when administering hydrophobic medicines that have limited solubility in aqueous environments. Given the special qualities of hydrogels, this delivery method presents itself as a viable means of forming hydrophobic medicines that are poorly soluble. With this method, medicinal substances can be administered orally, topically, or parenterally to patients and researchers (23). Hydrogels, with their capacity to alter form, porosity, surface morphology, & size, have opened up new avenues for addressing diverse issues in local therapies. These features allow hydrogels to be investigated for a wide range of biological and biomedical applications, including delivery of drugs, tissue engineering, controlled release of drugs, and so on (24).

Therefore, this work investigated the potential preventative effects of broccoli (*Brassica oleracea* var. *italica*) extract-based Hydrogel obtained from different phenological stages in conjunction with its bioactive component SFN for the treatment of breast cancer. The extract and Hydrogel both were characterized, and the *in vitro* and *in vivo* studies were done to check the anti-breast cancer activity. For *in vitro* studies the MCF-7 (Breast Cancer) and 3T3-L1 (Mouse Fibroblast cell line) cell lines were used. For *in vivo* Female BALB/c mice were used.

## 2 Experimental section

### 2.1 Chemicals required

Broccoli Extract (Prepared in laboratory), Fibroblast cells were used as the control group, and the selection of MCF-7 breast cancer cells was based on the preservation of multiple characteristics common to the mammary epithelium. Both cell line culture was purchased from National Center for Cell Science (NCCS) Pune, India. Sulforaphane was purchased from Sigma Aldrich, Carbopol 940 (Sigma Aldrich Karnataka, India), propyl paraben (Loba Chemicals Maharashtra, India), methyl paraben (Loba Chemicals Maharashtra, India), propylene glycol (Sigma Aldrich Karnataka India), triethanolamine (Sigma Aldrich, Karnataka, India), distilled water, Methanol (Himedia Maharashtra, India), Ethanol (Himedia Maharashtra, India), Female BALB/c Albino mice (ISF College of Pharmacy, Moga, Punjab), DMBA ((7,12-dimethylbenz[a]anthracene) (Sigma Aldrich Karnataka India), Deep freezer, Tissue homogenizer, Thiobarbituric acid (TBA), Tris HCl (Loba Chemicals Maharashtra India), Trichloroacetic acid, Centrifugation machine, Ellman’s reagent, PBS, Griess reagent, EDTA, 4% paraformaldehyde, Paraffin, hematoxylin and eosin Stain. All other reagents were purchased of analytical grade.

### 2.2 Formation of broccoli extract based hydrogel

Preparation of Broccoli Extract and Broccoli extract-based Hydrogel with all the characterizations and *In vitro* studies has already been published in my previous literature [25 (A) and 25 (B)] and *in vivo* studies will be assessing in this research paper.

### 2.3 Hemolysis study of prepared extract and formulation

The *in vitro* assessment of hemolytic activity is a widely used and essential approach for the initial evaluation of the cytotoxic effects of chemicals, pharmaceuticals, or materials that come into contact with blood, including medical devices.

The assay included varying concentrations (5, 10, 15 and 20 μg/mL) of Broccoli extract and Hydrogel. Hemolysis values below 10% are categorized as non-hemolytic, whereas values exceeding 25% are indicative of hemolytic potential. Two control samples were prepared without the samples: the negative control was treated with sterile phosphate-buffered saline, while the positive control was treated with 0.1% Triton X-100. The mean values were obtained from three independent experimental replicates. Triton X-100, serving as the positive control, induced complete hemolysis (100%), while PBS as the negative control showed minimal hemolysis. (26).

### 2.4 Cellular uptake (internalization) studies

Another important step in the formulation-making process is cell internalization, which is the process by which particle-encapsulated biomolecular drugs enter cells by endocytosis, adhesion, diffusion, or direct penetration.

Cellular Absorption of Prepared Broccoli Extract, Plain Drug, and with minor adjustments, confocal microscopy was used to study the formulation. For this, 24 & 48 hours before the experiment, MCF-7 cells were sown in plates. To study the internalization, cells were treated with 5 μM/mL of FITC labeled drug solution and incubated for 24 & 48 hr. After removing the unentrapped residue with three PBS washes (0.2 M, pH 7.4), the cells were photographed using a confocal microscope set to 488 nm for excitation and 515 nm for long pass filtering to detect emission light. The pictures were processed, and the formulations’ cellular absorption was examined. The procedure followed previously used by 27 and 28.

### 2.5 *In vivo* evaluation of broccoli extract and developed formulation

#### 2.5.1 Procurement and adaptation of mice to laboratory conditions

To evaluate the anti-cancer activity of broccoli extract and formulation, 48 adult Female BALB/c Albino mice, aged 5–6 weeks, weight 24.5 ± 0.5 g were purchased from ISF College of Pharmacy, Moga, Punjab in the year 2025, India. Mice were kept in animal house in groups of six per cage. They were kept in controlled environments with a 12-hour light/12-hour dark cycle, an ambient temperature between 22 and 25 °C, and humidity levels kept between 50 and 55%. Furthermore, food and drinking water were freely available to the mice. The animals were given a seven-day acclimatization time to become used to the lab environment before the experiment started. The Committee for Control and Supervision of studies on Animals (CCSEA) criteria were adhered to when conducting the animal studies. The ethical approval for this study was duly obtained from the Institutional Animal Ethical Committee with an approval number of ISFCP/IAEC/CCSEA/Meeting No 07/2025/Protocol No 86 at ISF College of Pharmacy, Moga, Punjab, India.

#### 2.5.2 Grouping of animals and induction of BC

**Figure 1:**
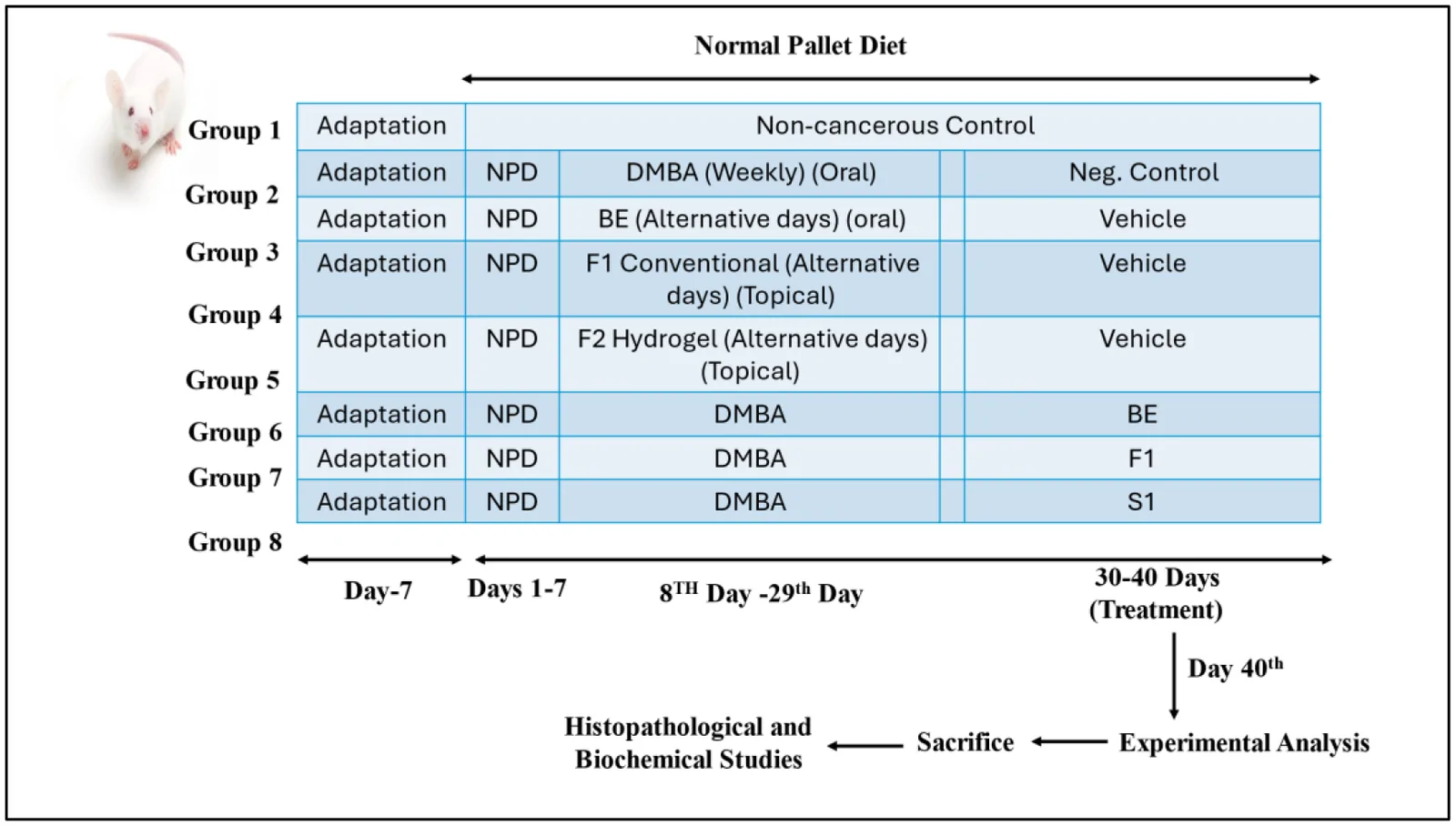
Schematic representation of the experimental design for evaluating the therapeutic effects of BE and Formulation in DMBA induced mice model.

After a one-week acclimatization period, all the mice were given NPD (Normal Pellet Diet), for 7 days, on the 8^TH^ day the all the animals were divide in 8 groups, 6 animals in each group, group 1 was served as the positive control group (Non-cancerous), this group was kept on NPD throughout the study, group 2 was served as Negative Control (DMBA Induced). DMBA (7,12-dimethylbenz[a]anthracene) was induced weekly by oral dosing. Mammary tumors in Female Albino mice (BALB/c) were developed as previously described by 29. Every day, the morbidity & mortality of the animals were assessed. Experimental design is shown in **Figure 1**.

#### 2.5.3 Assessment of body weights in DMBA infused mice

All groups of animals were documented at the end of the study for the body weight analysis. An appropriately adjusted electronic balance was used for measuring body weight of experimental animals (30).

#### 2.5.4 Biochemical estimation

Serum lipid profiles and other biochemical parameters were determined as part of the biochemical estimates. measurements of a number of chemicals in biological samples, such as serum, including indicators like creatinine, BUN, and uric acid and enzymes like ALT, AST, and ALP, in order to evaluate physiological state (31). The biochemical estimations included measuring the activity of lipid peroxidation, hydroperoxides, glutathione, antioxidant vitamins, and many enzymes, including lactate dehydrogenase, alkaline phosphatase, aspartate transaminase, and alanine transaminase (32).

#### 2.5.5 Tissue preparation

Animals in each group were sacrificed for biochemical estimations, histological assessment. All the tested samples were run in duplicate to minimize statistical errors. Following animal sacrifice, the livers were separated and kept cold (-80 °C) until they could be examined. The liver samples were taken out of the deep freezer, weighed, and mixed in 0.1 M phosphate buffer (pH 7.4) on the day of the experiment. Biochemical estimates were then performed using the homogenized striatal suspension solution.

#### 2.5.6 Estimation of lipid peroxidation

In short, two hours were spent incubating 0.5 mL of supernatant with 0.5 mL Tris HCl at 37 °C. One milliliter of 10% trichloroacetic acid was then added after incubation, and the mixture went through a centrifuge for ten minutes at 10,000 g. Following the collection of the resultant supernatant, 1 mL of 0.67% thiobarbituric acid was added to further dilute it. Finally, the tubes spent ten minutes in boiling water. One milliliter of water that had been double-distilled was added once it had cooled, and absorbance was measured at 532 nm using a spectrophotometer (UV-1700 Pharma Spec, Shimadzu, Japan). Thiobarbituric acid reactive substances (TBARS) were quantified using an extinction coefficient of 1.56 × 105 M^−1^ cm^−1^ and expressed as nmol of malondialdehyde (MDA) per mg protein (33).

#### 2.5.7 Estimation of reduced glutathione

The amount of reduced glutathione in the liver sample was determined using the Ellman method. This procedure involved cold digesting 1 mL of supernatant at 4 °C for 1 hour after precipitating it using 1 mL of 4% sulfosalicylic acid solution. At 4 °C, the sample was centrifuged at 12,000 g for 15 minutes. 1 mL of this supernatant was mixed with 2.7 mL of a buffered phosphate solution (0.1 M, pH 8) & 0.2 mL of 5,5-dithiobis (2-nitrobenzoic acid). The yellow color was measured using a spectrophotometer, that had appeared right away at 412 nm (34).

#### 2.5.8 Estimation of nitrite concentration

To assess nitrite buildup, a colorimetric test based on the Griess reagent—which consists of 0.1% N-(1-naphthyl) ethylenediamine dihydrochloride, 1% sulphanilamide as well as and 2.5% phosphoric acid—was carried out in the culture supernatant, which serves as an indicator of nitric oxide (NO) production. Following a 10-minute incubation period at room temperature in a dark environment, equal quantities of the reagent and supernatant were combined. The absorbance of the resulting solution was then recorded at 540 nm using a spectrophotometer. Nitrite concentrations were quantified by referencing a standard curve prepared with sodium nitrite (35).

#### 2.5.9 Estimation of superoxide dismutase activity

The test system included 96 mM nitro blue tetrazolium, 50 mM sodium carbonate, & 0.1 mM EDTA. Two milliliters of the aforementioned mixture, 0.05 milliliters of hydroxylamine, and 0.05 milliliters of the supernatant have been transferred to the cuvette in order to quantify the auto-oxidation of hydroxylamine. To test the absorbance at 560 nm, a Perkin Elmer Lambda twenty UV spectrophotometer was used (Himedia Laboratories, Mumbai, India) at 30-second intervals (36).

#### 2.5.10 Tissue isolation and histopathological assessment of liver and breast

At the end of the experiment, the breast and liver tissues were isolated from control, cancerous, and treated groups. Samples were preserved in 4% paraformaldehyde for a period of 24 hours, embedded in paraffin, & sectioned at 5-μm intervals for histological processing. Histopathology of the breast tissue and liver will be done where the breast tissue will be fixed in 10% v/v formal saline and embedded in paraffin. 5µm transverse section will be prepared and mounted on a slide coated with poly L-Lysine and will be stained with hematoxylin and eosin for viewing glomerular space and tuft area while picrosirius red dye will be used to check extracellular matrix deposition. Organs weights will also be taken at the termination of the study. The stained slices were examined using a fluorescence microscope (Model: 102 M, Motic Microscopes, China) at ×40 magnification (37).

### 2.6 Statistical analysis

All experiments data were obtained from three independent experiments. Values are presented as mean ± SD (n = 3). Statistical significance is indicated as ###p < 0.005 compared to normal control, and ***p < 0.005 compared to Triton X-100 (positive control).

## 3 Results

### 3.1 Hemolysis activity of extract and formulation

The hemolytic activity of the sample was tested under *in vitro* conditions, for each sample, various concentrations were added to 0.85% NaCl solution and then received a 2% suspension of human erythrocytes. After 30-min incubation at room temperature, cells were centrifuged and the supernatant was used to measure the absorbance of the liberated hemoglobin at 540 nm.

**Figure 2:**
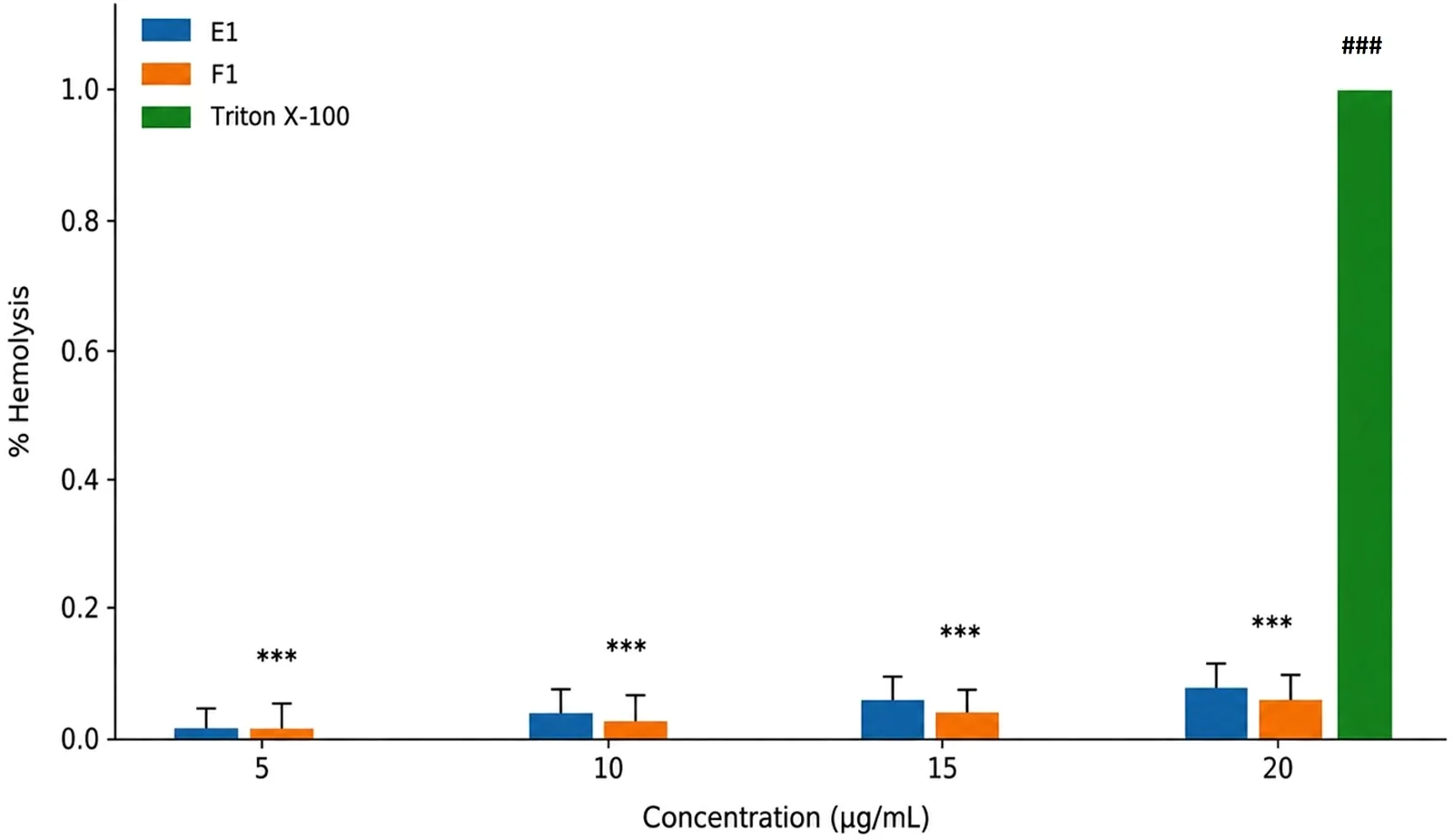
Represents % of hemolysis after treatment with E1 (Broccoli Extract) and F1 (Formulation) at varying concentrations 5, 10, 15 and 20 µg/mL. Values are presented as mean ± SD (n = 3). Statistical significance is indicated as ###p < 0.005 compared to normal control, and ***p < 0.005 compared to Triton X-100 (positive control).

All the groups were observed to be light yellow, which was similar to the negative control (PBS) group, while the Triton X-100 group was bright red. At the highest concentration both extract and Hydrogel exhibited a very low hemolysis (1.3 ± 0.05% and 1.2 ± 0.04%) indicating the best hemocompatibility as shown in **Figure 2**.

### 3.2 Cellular uptake (internalization) studies

**Figure 3:**
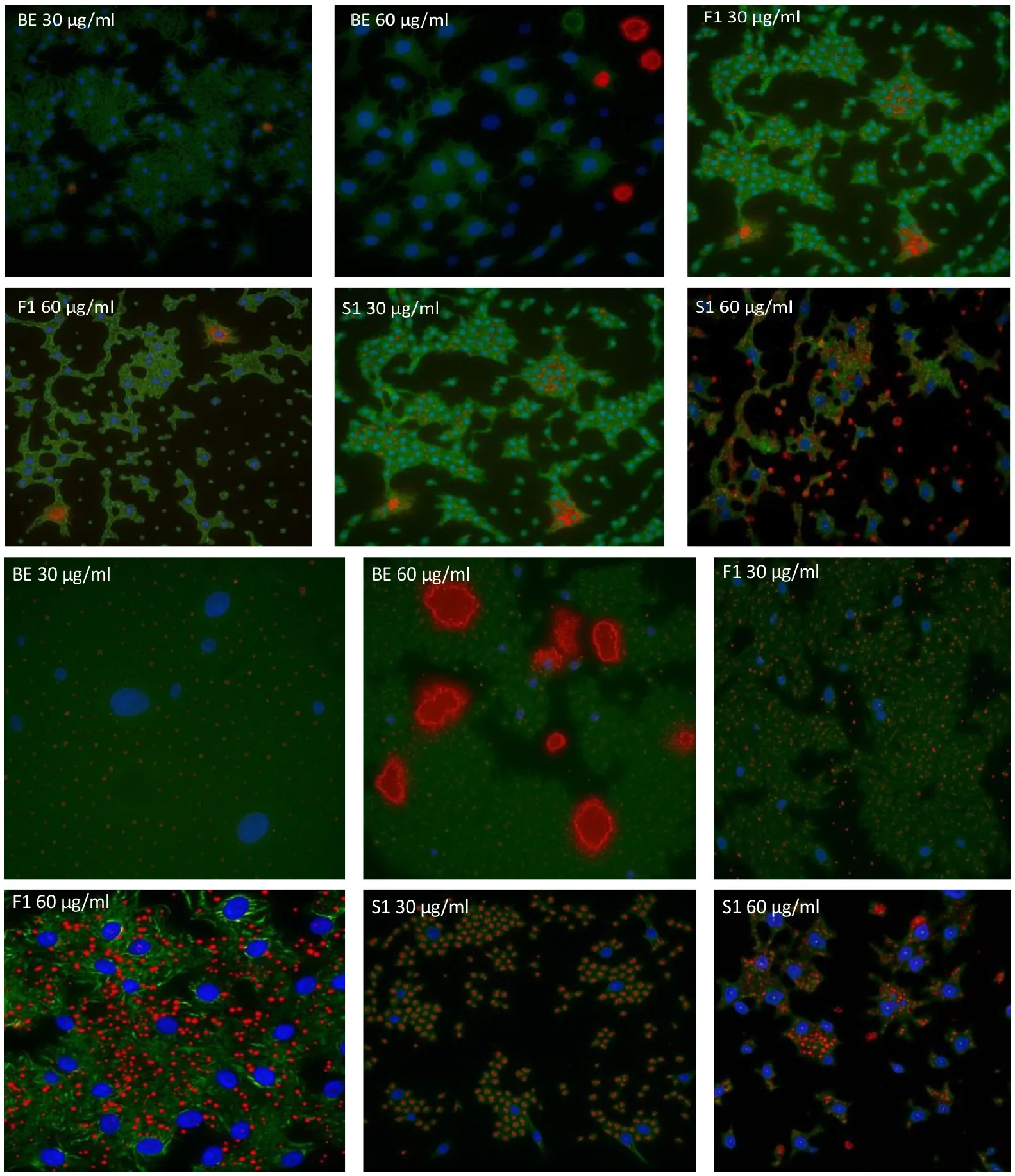
**(A)** Illustrates the cellular uptake (internalization) of BE, F1, and S1 formulations by MCF-7 cells after 24 hours of incubation at two different concentrations. The figure highlights the extent to which each formulation is internalized by the cancer cells, providing insights into their potential for targeted delivery and therapeutic efficacy. **(B)** depicts the cellular uptake (internalization) of BE, F1, and S1 formulations by MCF-7 cells following 48 hours of incubation at two different concentrations. This figure provides comparative insight into the time-dependent internalization efficiency of each formulation, reflecting their potential for sustained intracellular delivery and enhanced therapeutic performance over extended exposure.

In order to investigate the internalization of prepared broccoli extract, Formulation and Plain drug the studies were conducted at the two-time incubations i.e., 24 and 48 hrs. For this, BE, F1 & S1 were embedded in MCF-7 cells at 30 µg/ml and 60 µg/ml of concentrations till the above-mentioned time intervals. Confocal microscopy was used to examine the qualitative assessment of cell uptake. The BE, F1, and S1 were tagged with rhodamine, and the cell’s nucleus was stained with DAPI. **Figure 3B** illustrates that internalization of BE, F1, and S1 was noted after 24 hours, whereas significant cellular uptake was attained after 48 hours. As shown in **Figures 3A, B** BE, F1 & S1 are distributed within the cytoplasm near the nuclear boundary.

### 3.3 *In vivo* results

#### 3.3.1 Administration of broccoli extract and hydrogel mitigates body weight gain in DMBA induced cancerous mice

**Figure 4:**
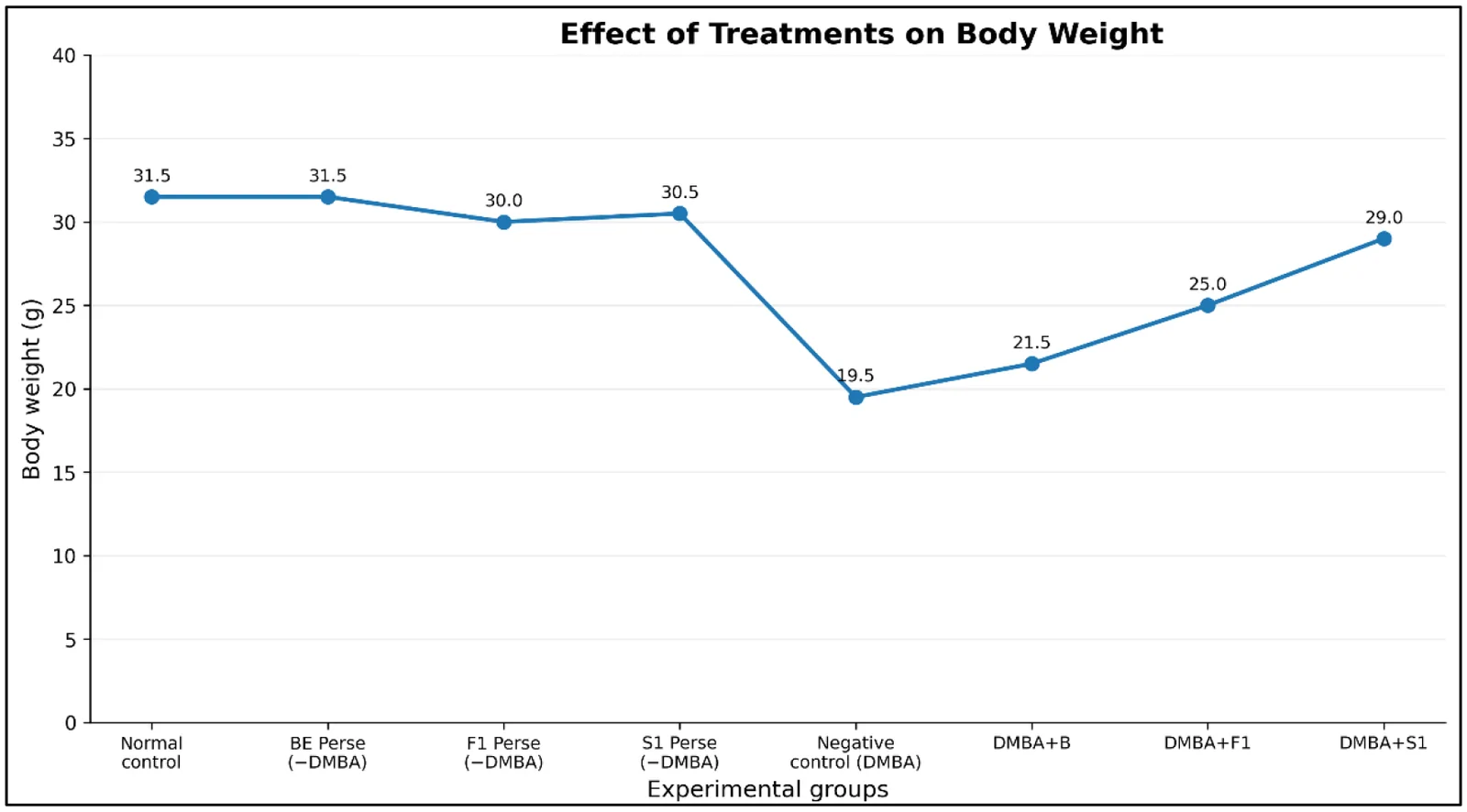
Effect on body weight (g). Data are shown as mean ± SD. ^@^p vs. Normal control group, F1, F2, and BE Perse, $p vs. Negative control group, and #p vs. DMBA+F1 group. The results were evaluated using one-way ANOVA, followed by Tukey’s *post hoc* analysis, where ^***^p<0.001, ^**^p<0.01, ^*^p<0.05 ^ns^P, not significance.

The effect of E1, F1 and S1 treatment on body weight was monitored at the end of the experimental period. As shown in **Figure 4**, untreated cancerous mice induced by DMBA exhibited a significant decrease in body weight (p < 0.001) compared to normal control mice fed NPD. Treatment with BE (400mg/Kg), F1(400mg/Kg) and S1 (50mg/Kg) significantly attenuated body weight gain (p<0.01 and p<0.05 respectively). These results collectively indicate that F1 treatment, particularly at 400 mg/Kg, effectively increases the body weight in DMBA induced mice. F1, E1 and S1 treatment with this concentration showed no discernible toxicity, as evidenced by no appreciable changes in body weight in perse (without DMBA induced) mice groups.

#### 3.3.2 Oxidative stress parameters

#### 3.3.3 Administration of broccoli extract and hydrogel decrease the level of MDA (oxidative stress parameter) in DMBA induced cancerous mice

**Figure 5:**
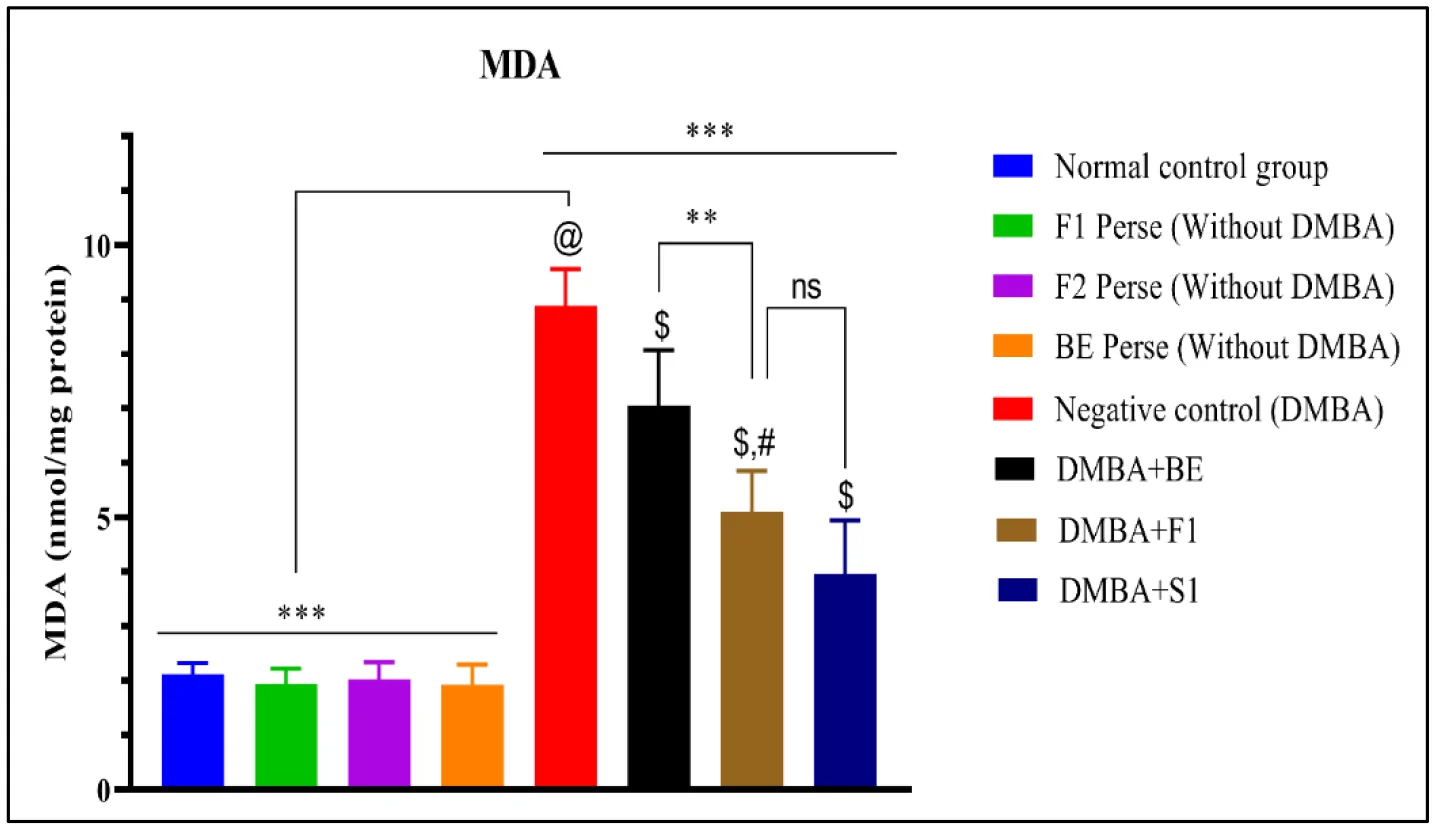
Effect on MDA levels in animal tissue samples. Data are shown as mean ± SD. ^@^p vs. Normal control group, F1, F2 and BE Perse, $p vs. Negative control group, #p vs. DMBA+BE group. Tukey’s *post hoc* analysis was performed after a one-way ANOVA was used to assess the findings, where ^***^p<0.001, ^**^p<0.01, ^ns^P = not significance.

According to the current study, administering DMBA raised oxidative stress in the liver cells by raising MDA levels and lowering antioxidant enzyme levels. The negative control showed the increased level of MDA (9 nmol/mg Protein) comparative to the control group. Our results showed that under non-disease conditions, MDA levels were reduced, whereas a significant increase in this marker was observed in the DMBA-induced cancer group (negative control). Administration of BE, F1, and S1(400mg/Kg, 400mg/Kg and 50mg/Kg) resulted in a notable reduction in MDA levels (7 nmol/mg, 5.5 nmol/mg and 4.9 nmol/mg Protein respectively) compared to the negative control (p<0.001 and p<0.01 respectively). Although all treatments exhibited significant antioxidant effects, a more pronounced reduction was observed with S1, followed closely by F1, indicating the strong antioxidant potential of the formulation. These findings suggest that while broccoli extract (BE) alone possesses antioxidant activity, the developed F1 derived from BE demonstrated superior efficacy, highlighting its enhanced antioxidant potential as shown in **Figure 5**.

#### 3.3.4 Antioxidant enzyme parameters

##### 3.3.4.1 Restoration of the GSH (glutathione reductase) by administration of broccoli extract and hydrogel in DMBA induced cancerous mice

**Figure 6:**
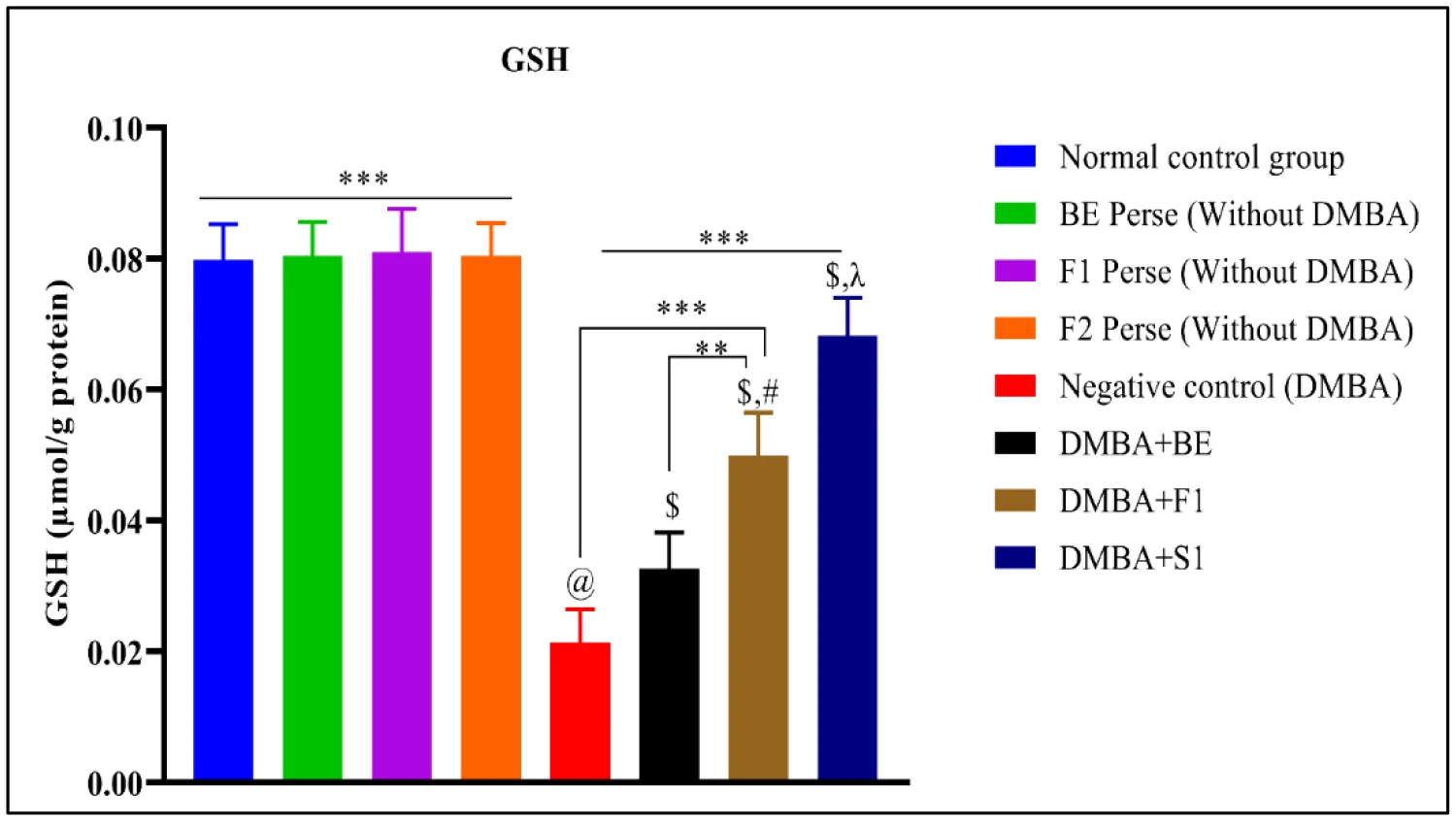
Effect on GSH levels in animal tissue samples. Data are shown as mean ± SD. ^@^p vs. Normal control group, F1, F2 and BE Perse, $p vs. Negative control group, #p vs. DMBA+BE group, ^λ^p vs. DMBA+F1. One-way ANOVA followed by Tukey’s *post hoc* test, where ^***^p<0.001, ^**^p<0.01.

A common tripeptide that is biosynthesized *in situ* at high quantities, glutathione (GSH) regulates cellular homeostasis through a variety of methods. GSH’s antioxidant ability, which supports the maintenance of cells’ redox cycle, is its primary known function. GSH peroxidases contribute to the scavenging of several types of ROS and RNS in order to achieve this. In this study, it was seen that GSH levels were decreased to (0.02 µmol/g Protein) in the DMBA induced mice as compared to the normal (non-diseased) and BE & F1 (Perse group). When treated with BE, F1 & S1, there was significant increase (0.03 µmol/g, 0.05 µmol/g & 0.07 µmol/g Protein respectively) in the GSH levels (p<0.001 & p<0.01 respectively), which shows the potent antioxidant effect of the Formulation in DMBA induced mice as shown in **Figure 6**. Hence the developed formulation is having a potent effect in lowering the oxidative markers and increasing the antioxidant ability of the cells.

##### 3.3.4.2 Administration of broccoli extract and hydrogel enhances the SOD (superoxide dismutase) activity in DMBA induced cancerous mice

**Figure 7:**
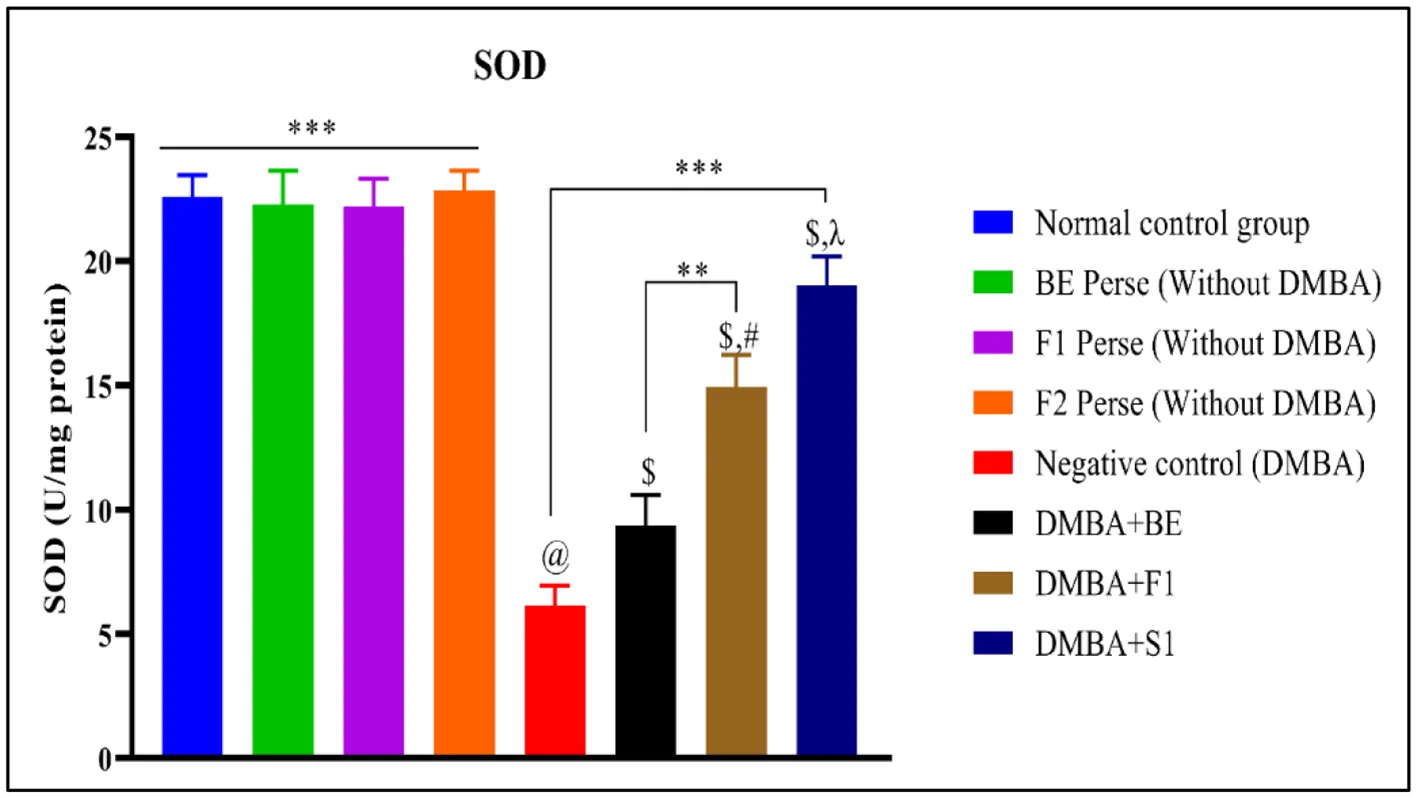
Effect on SOD levels in animal tissue samples. Data are shown as mean ± SD. ^@^p vs. Normal control group, F1, F2 and BE Perse, $p vs. Negative control group, #p vs. DMBA+BE group, ^λ^p vs. DMBA+F1. One-way ANOVA followed by Tukey’s *post hoc* test, where ^***^p<0.001, ^**^p<0.01.

The metalloprotein known as superoxide dismutase (SOD) is the first line of defense against oxidative stress because it catalyses the conversion of superoxide radicals into hydrogen peroxide and oxygen. When it comes to shielding cells against lipid peroxidation, it is crucial. In the present study, as seen in **Figure 7**, DMBA caused oxidative damage in the liver, which consequently led to a reduction in the antioxidant enzyme SOD (6.5 U/mg of protein). SOD, on the other hand, markedly increased the levels of antioxidant enzymes in the BE, F1, and S1 groups (9.1 U/mg of protein, 14.2 U/mg of protein, & 18 U/mg of protein, respectively). Thus, the treatment groups’ increased levels of total protein demonstrated a notable improvement in hepatic cells due to the formulation’s protective effects.

##### 3.3.4.3 Administration of broccoli extract and hydrogel lowers the nitrite levels in DMBA induced cancerous mice

**Figure 8:**
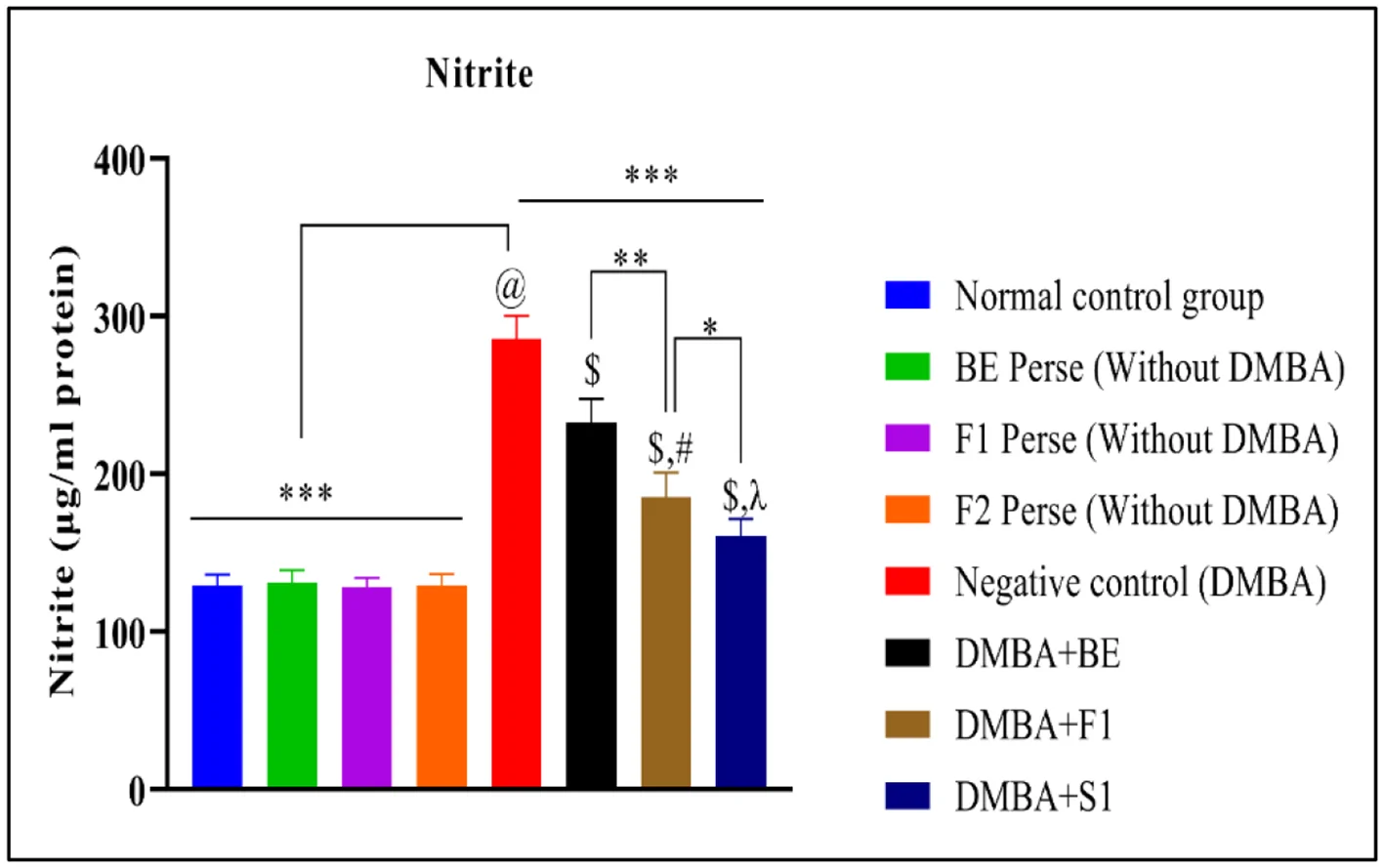
Effect on nitrite levels in animal tissue samples. Data are shown as mean ± SD. ^@^p vs. Normal control group, F1, F2 and BE Perse, $p vs. Negative control group, #p vs. DMBA+BE group, ^λ^p vs. DMBA+F1. One-way ANOVA followed by Tukey’s *post hoc* test, where ^***^p<0.001, ^**^p<0.01.

Patients with breast cancer, especially those in late stages, have been found to have elevated nitrite levels and other oxidative stress indicators. These results point to a possible involvement of nitrites & the nitric oxide pathway in the initiation and spread of breast cancer. In the DMBA induced group the Nitrite level was increased (290 µg/ml Protein) when compared with the control group, as presented in **Figure 8**. Further when treated with the BE, F1 & S1 there was decrease in the nitrite levels (230 µg/ml Protein, 185 µg/ml Protein & 170 µg/ml Protein respectively). By these results we can conclude that the developed formulation is very effective against the increased levels of Nitrite in DMBA induced mice.

### 3.4 Histopathological assessment of hepatic and breast tissues following broccoli extract and developed formulation

We conducted histopathological assessments of hepatic and breast tissues to evaluate the impact of BE & F1 administration. These tissues were selected due to their crucial roles in the metabolic and organ-specific effects of cancer. The following sections detail the histological changes observed in each organ following treatment with BE & F1, highlighting the potential protective effects and restoration of tissue architecture.

#### 3.4.1 Histological examination of hepatic tissues across experimental groups

**Figure 9:**
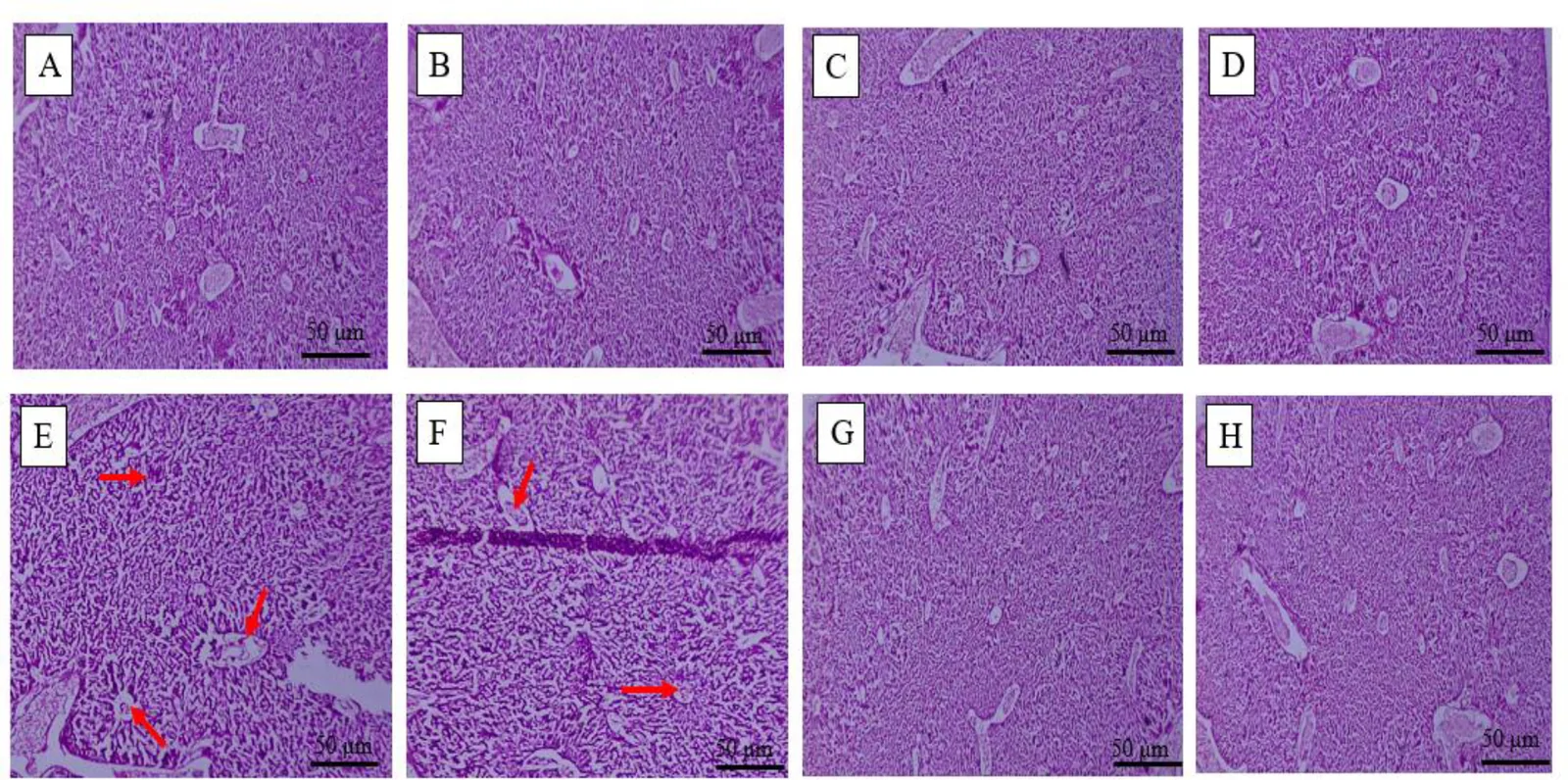
Histopathological analysis of liver tissues from different experimental groups stained with hematoxylin and eosin (H&E). **(A)** Normal control group, **(B)** BE perse (without DMBA), **(C)** F1 perse (without DMBA), **(D)** F2 perse (without DMBA), **(E)** Negative control (DMBA), **(F)** DMBA+BE, **(G)** DMBA+F1, **(H)** DMBA+S1, red arrow shows morphological changes with large globule and degradation fat in liver tissue sample.

The results pertaining to **Figure 9** reveal that the histopathological analysis of liver tissues showed marked differences among the experimental groups. The normal control group exhibited well-preserved hepatic architecture, with uniformly arranged hepatocytes, clear cytoplasm, prominent nuclei, intact sinusoidal spaces, and a well-organized central vein, indicating normal hepatic morphology. However, the cancerous control group showed severe hepatic injury, characterized by extensive hepatocellular degeneration, cytoplasmic vacuolization, sinusoidal dilation, inflammatory cell infiltration, and disruption of hepatic cords, reflecting significant structural disorganization due to the induction of breast cancer. Liver tissues displayed significant morphological alterations, including large lipid globules and degraded fat tissue. In contrast, groups treated with BE and its formulations (F1, F2, and S1) demonstrated marked restoration of normal histoarchitecture in liver tissues. Overall, these findings suggest that BE & F1 treatment, particularly at 400mg/Kg, significantly mitigates breast cancer-induced hepatic injury and promotes pronounced histological recovery in a dose-dependent fashion. The histological observations align with the biochemical findings and further validate the therapeutic efficacy of broccoli-based hydrogel in mitigating DMBA-induced carcinogenic and hepatotoxic effects.

#### 3.4.2 Histological examination of breast tissues across experimental groups

**Figure 10:**
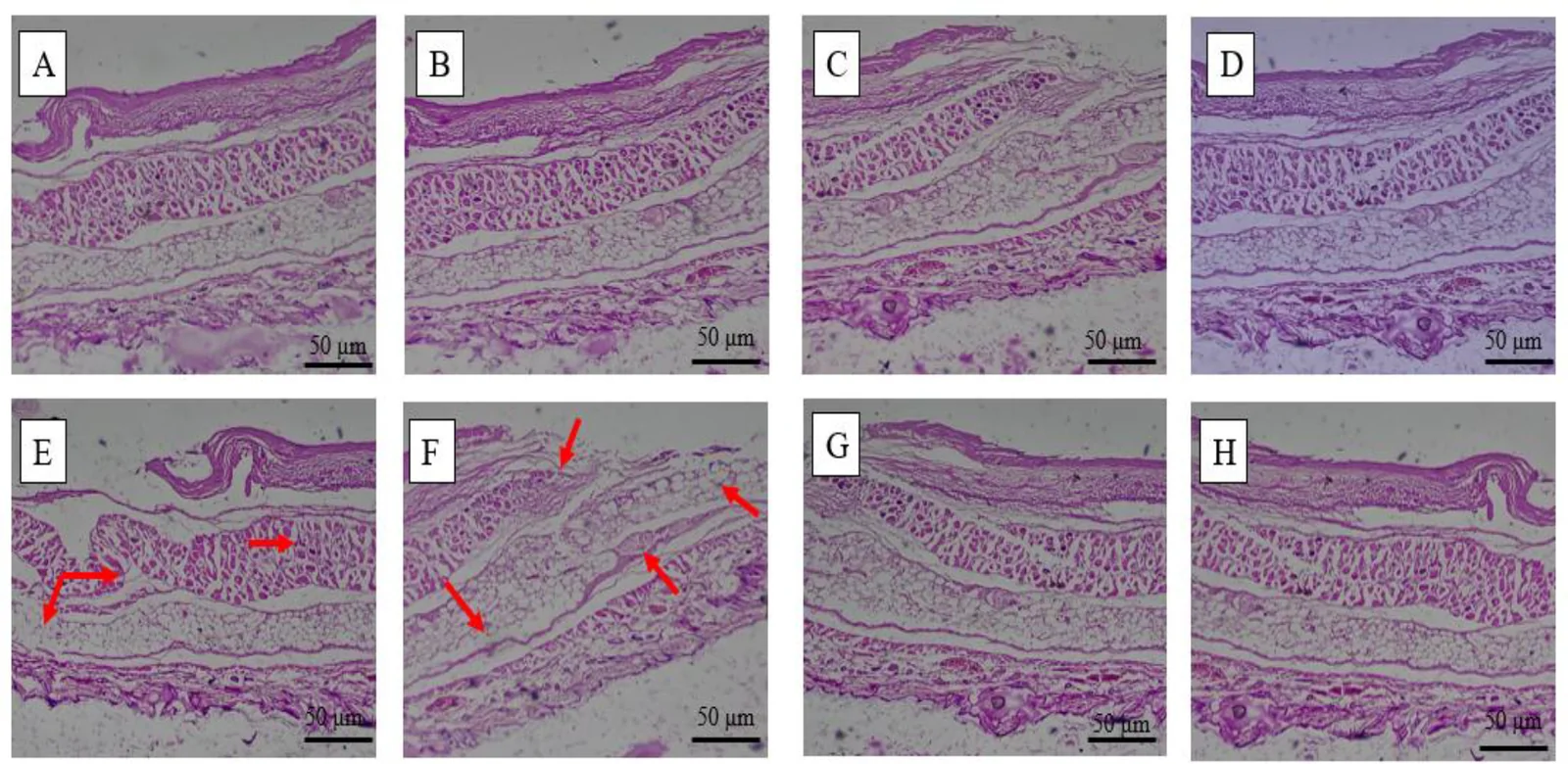
Histopathological examination of breast tissues from the experimental groups was carried out with hematoxylin and eosin stain (H&E). **(A)** Normal control group, **(B)** BE perse (without DMBA), **(C)** F1 perse (without DMBA), **(D)** F2 perse (without DMBA), **(E)** Negative control (DMBA), **(F)** DMBA+BE, **(G)** DMBA+F1, **(H)** DMBA+S1, red arrow shows small vessel congestion and inflammatory cell infiltration in tissue sample.

Histopathological analysis revealed that the control group’s mammary tissue had normal ductules and acini as shown in **Figure 10**. In the DMBA-treated negative control group, breast tissues showed pronounced small vessel congestion and inflammatory cell infiltration (indicated as red arrow), In contrast, groups treated with BE and its formulations (F1, F2, and S1) demonstrated marked restoration of normal histoarchitecture in breast tissues. Particularly, the S1 formulation showed reduced inflammatory infiltration and vascular abnormalities in breast tissue, along with preserved hepatic structure, indicating its superior protective potential. These histological findings are consistent with the biochemical results and further validate the therapeutic efficacy of broccoli-based hydrogel in mitigating DMBA-induced carcinogenic and hepatotoxic effects.

## 4 Discussion

According to the IARC, BC exceeds lung cancer in prevalence & is the leading cancer concern globally, making it the most common cancer among women in 2020. According to Zhang et al. (38), there are over 2.3 million novel cases diagnosed per annum, which represents 11.7 percent of all cancer cases. In the majority of countries, BC accounts for 16.7% of cancer-related fatalities and 17% of cancer diagnoses (5).

Currently available treatment options include hormone therapy, radiation, chemotherapy, and surgery. Additionally, immunotherapy, targeted therapy, & gene therapy are being used more and more. These developments have decreased mortality while greatly increasing life expectancy and quality. For instance, individuals with BC now have a much better prognosis because to the usage of inhibitors against PD-1 and PDL-1 (39). Drug resistance and BC recurrence, however, present serious difficulties, especially in the violent TNBC subtype, which shows little response to current hormonal & targeted treatments. Finding novel therapeutic targets and anticancer drugs is therefore crucial (40).

Natural products have great potential for contemporary medication development because of their diverse biological activity, large range of sources, and structural variety (41). However, issues like low stability and bioavailability prevent natural products from being used clinically to treat breast cancer. Overcoming these constraints may be possible with the use of cutting-edge technologies like nanoformulations. In order to overcome these obstacles, novel drug delivery systems (DDSs) have become essential instruments for improving drug stability, extending circulation, and permitting tumor-specific release (42). However, the majority of DDSs encounter translational obstacles: Because of stromal constraints and ECM remodeling, less than 5% of systemically given doses reach tumor sites, and clinical adoption is delayed by scalability problems and insufficient *in vivo* screening technologies (43).

Among DDSs, hydrogels are particularly adaptable platforms because of their biocompatibility, stimuli-responsive behavior, and adjustable physicochemical characteristics (43). Hydrogels offer a viable foundation for both local and long-term treatment among other approaches. Hydrophilic gels are often made of biocompatible polymers, either synthetic or natural. Interestingly, hydrogels possess inherent mechanical, physicochemical, and physical characteristics that allow for the encapsulation of various agents, including nanomaterials and chemotherapeutics, which facilitate the directed and prolonged release of these agents to the intended application site (44).

Broccoli, or *Brassica oleracea* is one of the most popular vegetables. It has a high content of soluble fiber and a variety of nutrients, such as vitamins A, C, (45). Because broccoli (*Brassica oleracea*) naturally has a high quantity of phytochemicals that are bioactive such glucosinolates, phenolic components, vitamin C, and mineral elements, it has been advertised as a food that promotes health. Therefore, eating a significant amount of broccoli contributes to the prevention of chronic illnesses like cancer, cardiovascular disease, and carcinogenic pathologies. It also helps prevent breast and prostate cancer (46). According to reports, 25% of all cancers in women worldwide are breast cancers. This makes them the most common cancer in this population (47).

To cure BC, the broccoli extract was added to a hydrogel. To create a safe and stable gel, natural and semisynthetic ingredients are used in the formulation process. The hydrogel was made using varying concentrations of the gelling ingredient, and their pharmacological effects were examined. This broccoli extract based topical hydrogel formulation may be an advantageous tool for the efficient management and treatment of breast cancer.

After the *in vitro* studies the as published in our previous research Cellular uptake (Cell internalization) studies were done, as cellular uptake is the crucial step when it comes to the drug delivery. Following a 4-hour incubation period with MCF-7 cells, cellular uptake studies were conducted at two-time intervals of 24 and 48 hours. The results were aligned with the study conducted by 48, which assessed the effectiveness of the active targeting strategy. For this study, CLSM (Confocal Laser Scanning Microscopy) was utilized to visualize the uptake of both functional and non-functionalized SLN. When internalization studies were performed with the prepared extract, formulation and plain drug, at two different concentrations, it was seen that the confocal microscopy images illustrate the cellular uptake of three different formulations—BE, F1, and S1—at two concentrations (30 µg/mL & 60 µg/mL) in MCF-7 BC cells. Blue staining marks the nuclei (DAPI), green indicates the cytoskeleton or cell structure, and red fluorescence represents the internalized formulation. At 30 µg/mL, BE shows minimal red fluorescence, indicating low cellular uptake, while F1 displays a moderate increase in red signal, suggesting better uptake than BE. S1, even at 30 µg/mL, shows stronger and more widespread red fluorescence, implying superior uptake efficiency. At the higher concentration of 60 µg/mL, all formulations exhibit enhanced uptake, with BE showing larger red aggregates (possibly extracellular), F1 demonstrating dense red puncta throughout the cytoplasm, and S1 displaying the most intense and uniformly distributed red fluorescence localized near the nucleus. Overall, cellular uptake is dose-dependent, and the uptake efficiency across formulations follows the order: S1 > F1 > BE, highlighting S1 as the most effective formulation for intracellular delivery in MCF-7 cells again the outcomes were well-suited with the study of 49.

The present study highlights the promising therapeutic potential of broccoli extract-based hydrogel formulation in the treatment of breast cancer. Given the limitations associated with conventional chemotherapeutic agents-including high cost, systemic toxicity, and interference with normal metabolic processes, there is a critical need for safer, more affordable alternatives. Our study findings have previously demonstrated the significant antiproliferative activity of broccoli-derived bioactive phytochemicals on BC cells. We created an *in vivo* investigation employing a DMBA-induced breast tumor model developed in female albino mice (BALB/c) in order to confirm these findings.

The hydrogel formulation served as an efficient drug delivery system, overcoming the solubility limitations of hydrophobic compounds commonly encountered in natural extracts. Its biocompatibility, minimal toxicity, and favorable swelling behavior allowed for effective administration and sustained release of the therapeutic phytochemicals. However, the treatment groups, particularly BE, F1, and F2, significantly mitigated DMBA-induced weight loss, indicating a protective effect against DMBA-induced cancer. Notably, the DMBA+S1 demonstrated a significant improvement over the DMBA+F1 control. Similar outcomes were observed by 50, showing that liposome-encapsulated drugs, when used with hydrogel, may serve as an effective strategy for BC therapy to both significantly lower the death rate of mice and delay the growth of tumors, and The test mice did not exhibit a decline in animal weight because the polymeric hydrogel combined with IMQ-containing liposomes did not significantly harm them. The healthy state of tumor-bearing mice was associated with their body weight. For the treated mice, a significant body weight fluctuation was observed.

Additionally, the formulation showed a marked inhibition of tumor, reduced oxidative stress parameters like MDA and nitrite with restoration of GSH and SOD indicate the protective effect of BE, F1 and S1. These biochemicals play critical role in regulating oxidative stress and cellular loss. In DMBA treated animals the level of oxidative stress markers was enhanced as confirmed from increase MDA and nitrite level. The observed results confirm the enhanced efficacy and bioavailability of released formulation.

In agreement with previous reports, our data confirm a profound oxidative environment in liver, kidney, pancreas, and heart tissues of cancerous mice. Treatment with broccoli extract and Formulation notably ameliorated oxidative stress. Broccoli extract and developed formulation improved antioxidant enzyme activity and reduced LPO, aligning with prior studies demonstrating its efficacy in restoring redox balance and reducing inflammation (51). The impact on immunity is another crucial component of cancer induction and therapy. The immune-progenitor cell organs and peripheral immune parameters make up the immunological aspect.

Lipid peroxidation (LPO) is a significant indication of oxidative stress brought on by reactive oxygen species (ROS). A measure of LPO that rises in a number of illnesses, malondialdehyde (MDA) has been linked to DMBA-induced toxicity (52 & 53). The results of this investigation were consistent with 54, which showed that DMBA intoxication increased MDA in the liver, kidney, and brain, are in line with each other. Oxidative stress is caused by DMBA’s metabolism, resulting in reactive metabolites that generate free radicals, ultimately producing an oxidative imbalance with oxidants prevailing over antioxidants (55).

The DNA or protein cell cycle is harmed by ROS, which can lead to unchecked cell division and proliferation as well as the onset of cancer. Thus, the current study evaluated the preventive function of broccoli extract and developed hydrogel and the harmful effects of DMBA in several tissues. One oxidative stress biomarker that was reduced in liver & kidney of mice treated with DMBA as opposed to the control group was GSH (56). DMBA reduced the levels of GSH in the kidney (57), liver (58) and brain (59). It’s crucial to keep in mind that GSH is a non- protein thiol that helps scavenge the electrophilic molecules that cause and worsen a number of diseases. (60, 61).

These findings are steady with other research (62 & 63), that highlighted the antioxidant properties of broccoli extracts by lowering oxidative stress and boosting GSH concentrations, critical for scavenging lipid peroxides, in humans with type 2 diabetes and in diabetic rats. It was also observed that colon cell lines were protected from benzo(a)pyrene-induced DNA strand damage by SFN exposure (64). Generally speaking, brassica extracts and their constituents support DNA protection and antioxidant activities against many carcinogens (65).

As SOD is another important antioxidant enzyme, so it was seen in the present study, that the BE and F1 treatment restored the levels of the SOD enzyme in DMBA induced mice, and the fundings were similar to the previous study done by the 66. Further the results were consistent with another study by 67, in which It has been suggested that consuming broccoli extract and soy isoflavones together may help reduce the clinical symptoms of endometriosis.

Furthermore, histopathological evaluation of breast and liver tissues provided critical insights into the protective effects of the broccoli extract (BE) and its hydrogel formulations. The control rats had normal liver architecture and histology, with intact hepatocytes radiating from a normal-sized central vein with intact blood sinusoids and normal rounded euchromatic nuclei (68). In the DMBA-treated negative control group, breast tissues showed pronounced small vessel congestion and inflammatory cell infiltration (indicated as red arrow), while liver tissues displayed significant morphological alterations, including large lipid globules and degraded fat tissue. Histological analysis revealed alterations in liver morphology linked to hepatic lipidosis over time in DMBA induced mice and treatment with BE and F1 decreased lipidosis, results were connected with the previous study of (69).

Histopathological alterations in the mammary gland were examined using H&E staining. The results demonstrated that SFN effectively prevented the impact of LPS stimulation, which caused a high number of neutrophils to infiltrate the mammary acinar cavity, and the results are consistent with the findings of 70. In contrast, groups treated with BE and its formulations (F1, F2, and S1) demonstrated marked restoration of normal histoarchitecture in breast tissues. Particularly, the S1 formulation showed reduced inflammatory infiltration and vascular abnormalities in breast tissue, along with preserved hepatic structure, indicating its superior protective potential. These histological findings are steady with the biochemical results and further validate the therapeutic efficacy of broccoli-based hydrogel in mitigating DMBA-induced carcinogenic and hepatotoxic effects with the similar findings of the (71, 72).

Overall, the findings support the hypothesis that broccoli extract, particularly when delivered via hydrogel, can serve as a potent and safe anti-cancer agent. Broccoli extract is rich in bioactive phytochemicals, particularly sulforaphane, a potent antioxidant and anti-carcinogenic compound known to temper various cellular pathways involved in cancer development. It exhibits anti-inflammatory, pro-apoptotic, and detoxifying properties that contribute to its chemopreventive potential.

## 5 Conclusion

One of the most common and physiologically varied cancers in the world, BC is responsible for a significant percentage of cancer diagnoses & deaths among women. One of the biggest causes of cancer-related death for women is still breast cancer, even after decades of study and improvements in detection and therapy. This research aimed to overcome the confines of conventional anticancerous therapy by developing a Broccoli extract-based Hydrogel, for the targeted & controlled transdermal delivery of drugs for targeted BC therapy. Therefore, in the presented study, the Hydrogel with broccoli extract embedded in it, is developed for the Transdermal Drug Delivery (TDDs) for the BC treatment. In light of these difficulties, researchers have recently focused a lot of emphasis on precision hydrogel technology.

Our study findings have previously demonstrated the significant antiproliferative activity of broccoli-derived bioactive phytochemicals on BC cells. To further validate these results, we designed an *in vivo* study using a DMBA-induced mammary tumor model in female albino mice (BALB/c). Overall, the findings support the hypothesis that broccoli extract, particularly when delivered via hydrogel, can serve as a potent and safe anti-cancer agent. Broccoli extract is rich in bioactive phytochemicals, particularly sulforaphane, a potent antioxidant and anti-carcinogenic compound known to modulate various cellular pathways involved in cancer development. It exhibits anti-inflammatory, pro-apoptotic, and detoxifying properties that contribute to its chemopreventive potential. Additionally, broccoli extract helps in reducing oxidative stress and inhibiting tumor cell proliferation, making it a promising natural agent for cancer therapy. However, further studies involving long-term toxicity, pharmacokinetics, and molecular pathway analysis are essential to translate this formulation into clinical applications.

## Data Availability

The original contributions presented in the study are included in the article/supplementary material. Further inquiries can be directed to the corresponding authors.
